# A Bacterial Quorum-Sensing Precursor Induces Mortality in the Marine Coccolithophore, *Emiliania huxleyi*

**DOI:** 10.3389/fmicb.2016.00059

**Published:** 2016-02-03

**Authors:** Elizabeth L. Harvey, Robert W. Deering, David C. Rowley, Abrahim El Gamal, Michelle Schorn, Bradley S. Moore, Matthew D. Johnson, Tracy J. Mincer, Kristen E. Whalen

**Affiliations:** ^1^Department of Marine Sciences, Skidaway Institute of Oceanography, University of Georgia, SavannahGA, USA; ^2^Department of Biomedical and Pharmaceutical Sciences, University of Rhode Island, KingstonRI, USA; ^3^Scripps Institution of Oceanography, University of California at San Diego, La JollaCA, USA; ^4^Biology Department, Woods Hole Oceanographic Institution, Woods HoleMA, USA; ^5^Marine Chemistry and Geochemistry Department, Woods Hole Oceanographic Institution, Woods HoleMA, USA

**Keywords:** infochemicals, algicidal compound, bacteria–phytoplankton interaction, HHQ, *Pseudoalteromonas*, *Emiliania huxleyi*, IC50, mortality

## Abstract

Interactions between phytoplankton and bacteria play a central role in mediating biogeochemical cycling and food web structure in the ocean. However, deciphering the chemical drivers of these interspecies interactions remains challenging. Here, we report the isolation of 2-heptyl-4-quinolone (HHQ), released by *Pseudoalteromonas piscicida*, a marine gamma-proteobacteria previously reported to induce phytoplankton mortality through a hitherto unknown algicidal mechanism. HHQ functions as both an antibiotic and a bacterial signaling molecule in cell–cell communication in clinical infection models. Co-culture of the bloom-forming coccolithophore, *Emiliania huxleyi* with both live *P. piscicida* and cell-free filtrates caused a significant decrease in algal growth. Investigations of the *P. piscicida* exometabolome revealed HHQ, at nanomolar concentrations, induced mortality in three strains of *E. huxleyi*. Mortality of *E. huxleyi* in response to HHQ occurred slowly, implying static growth rather than a singular loss event (e.g., rapid cell lysis). In contrast, the marine chlorophyte, *Dunaliella tertiolecta* and diatom, *Phaeodactylum tricornutum* were unaffected by HHQ exposures. These results suggest that HHQ mediates the type of inter-domain interactions that cause shifts in phytoplankton population dynamics. These chemically mediated interactions, and other like it, ultimately influence large-scale oceanographic processes.

## Introduction

In the marine environment, interactions between bacteria and eukaryotic phytoplankton are pervasive and drive oceanic biogeochemical cycles (Legendre and Rassoulzadegan, 1995), and can have consequences for both microbial communities (Bratbak and Thingstad, 1985) and structuring of marine food webs (Azam et al., 1983). Bacteria–phytoplankton interactions are complex, being both temporally variable (Danger et al., 2007) and species-specific (Fukami et al., 1997), and remain largely enigmatic. These interactions can be beneficiary, as bacteria and phytoplankton can support the growth of one another via the exchange or recycling of nutrients (Azam et al., 1983); however, the interaction can also be detrimental, resulting in phytoplankton morbidity or mortality (Mayali and Azam, 2004). The intricate relationships between these two kingdoms are often mediated via excreted compounds that can direct communication between the two organisms. Identifying these released compounds, and their influence on the population dynamics of both phytoplankton and bacteria, will enhance our understanding of the role of “infochemicals” on large-scale biogeochemical processes.

The marine genus *Pseudoalteromonas* constitutes 0.5–6.0% of bacterial species globally (Wietz et al., 2010), and has been found in seawater, marine sediments, and associated with marine eukaryotes (Bowman, 2007; Skovhus et al., 2007; Sneed et al., 2014). Secondary metabolites secreted by members of this genus fulfill a variety of ecological roles, including involvement in chemical protection, settlement, induction of metamorphosis in marine invertebrates, and commercially, as antifouling, antifungal agents (Bowman, 2007; Ross et al., 2014; Sneed et al., 2014). Moreover, *Pseudoalteromonas* species have been implicated in producing algal-lytic compounds that cause mortality in dinoflagellates (Skerratt et al., 2002; Kim et al., 2009), diatoms (Mitsutani et al., 2001), and raphidophytes (Lovejoy et al., 1998); however, in each of these cases the causative chemical compound is yet unidentified.

In order to gain a more mechanistic understanding of this chemically mediated phytoplankton mortality, we exposed the globally important coccolithophore, *Emiliania huxleyi* to cultures of *Pseudoalteromonas piscicida*. *E. huxleyi* plays a predominant role in oceanic carbon (Balch et al., 1992; Iglesias-Rodriguez et al., 2008) and sulfur (Simo, 2001) cycling; therefore, understanding the factors that mediate the population abundance and distribution of this algal species is important for predicting its role in marine nutrient cycling and global climate. Further, *E. huxleyi* has been shown to form complex relationships with bacteria. For example, it has been observed that the Roseobacter, *Phaeobacter gallaeciensis* maintains a mutualistic-turned-to-parasitic relationship with *E. huxleyi* dependent on the metabolic stage of the algae (Seyedsayamdost et al., 2011). While no direct relationship between a *Pseudoalteromonas* species and *E. huxleyi* has been established, Seymour et al. (2010) found that *Pseudoalteromonas haloplanktis* exhibited strong chemoattraction to dimethlysulfoniopropionate (DMSP), a sulfur compound abundantly produced by *E. huxleyi*. Additionally, bacterial OTUs, including *Pseudoalteromonas*, were positively correlated with coccolithophores and *E. huxleyi* abundance in samples from a temperate marine coastal site off Plymouth, UK (Gilbert et al., 2012). Thus, these two taxa do co-occur in the water column, and have opportunity to interact with one another.

Here, we report the isolation and identification of a potent algicidal compound excreted by *P. piscicida* with specificity for *E. huxleyi*. We found that the presence of *P. piscicida* resulted in mortality of *E. huxleyi*, and used bioassay-guided fractionation to identify the responsible chemical mediator(s) of this interaction. This research further reveals the importance of the bacterial exometabolome in inter-domain interactions, especially those that can have significant biogeochemical implications.

## Materials and Methods

### Culture Conditions and Crude Extract Preparation

A pure culture of *P. piscicida* was isolated from open ocean plastic debris in the North Atlantic (Mincer culture ID; A757; Whalen et al., 2015), cryopreserved in 10% sterile DMSO, and stored at -85°C prior to experiments. From these stocks, multiple ‘starter’ cultures were prepared by inoculating 8 mL of TSW media (1 g tryptone in 1 L of 75:25 seawater/MilliQ water) with 100 μL of cryopreserved culture, and then incubated at 23°C while shaking at 100 rpm for 3 days. After 3 days, 2 mL of ‘starter’ culture was used to inoculate seven, 1.5 L Fernbach flasks of TSY media (1 g tryptone, 1 g yeast extract, 75% seawater), the newly inoculated cultures were then grown at 100 rpm for 8 days at 23°C. On day 7, 20 mL (approximately weight = 7.8 g) of 1:1 mixture of sterile Amberlite^®^ XAD-7 and XAD-16 resin that had been extensively washed in organic solvent, dried, and autoclaved was added to each 1.5 L culture. Twenty-four hours later (day 8), the resin was filtered from the bacterial culture through combusted stainless steel mesh under gentle vacuum (<5 in Hg), desalted by rinsing with MilliQ water, pooled, and allowed to dry overnight at room temperature. Bacterial metabolites were eluted from the resin first in 800 mL of (1:1) methanol (MeOH):dichloromethane (DCM), followed by 800 mL of methanol. This crude extract was dried under vacuum centrifugation and stored at -85°C until testing in phytoplankton growth assays.

*Emiliania huxleyi* (Plymouth Algal Culture Collection: DHB624 and National Center for Marine Algae: CCMP374, CCMP379), *Dunaliella tertiolecta* (CCMP1320), and *Phaeodactylum tricornutum* (CCMP2561) were used in these experiments. All phytoplankton cultures were grown in 0.2 μm sterile-filtered, autoclaved seawater (FSW), enriched with f/2 (*P. tricornutum*) or f/2 -Si (all other species) media (Guillard, 1975). All cultures were maintained on a 12:12 h light:dark at 18°C, salinity of approximately 20 psu, and light intensity of 85–100 μmol photon m^-2^ s^-1^. Hereafter, these conditions will be referred to as general culturing conditions. Phytoplankton cultures were transferred every 7–10 days to maintain exponential growth. Unless specified, cultures were not axenic. Axenic cultures of *E. huxleyi* 624 were prepared by adding 1 mL of a dense *E. huxleyi* culture into 4 mL of f/2 -Si containing an antibiotic cocktail of penicillin G (3 mM), dihydrostreptomycin sulfate (0.2 mM), and gentamicin (0.5 mM). The culture was incubated under the general culturing conditions described above for 48 h, after which a 500 μL aliquot of the culture was transferred to fresh, antibiotic-free f/2 -Si media. This culture was maintained in exponential growth, similar to the other phytoplankton cultures. The culture was tested with DAPI (4′, 6-diamidino-2-phenylindole) to ensure it remained axenic throughout the course of the experiments, and this culture was used for all experiments that called for axenic *E. huxleyi*. For all phytoplankton counts, a 200 μL aliquot of culture was run on a flow cytometer (Guava, Millipore). Cell abundance was determined by using species-specific settings determined based on chlorophyll a (692 nm) and side scatter (SSC) for each species examined.

### Phytoplankton Growth Inhibition Assay

The growth rate of *E. huxleyi* (strain 624) was measured in response to both live *P. piscicida* cells (10^3^ and 10^6^ cell mL^-1^) and filtrate from *P. piscicida* cells equivalent to 10^3^ and 10^6^ cell mL^-1^ in TSY media (between <1 and 40 μL of filtrate per 1 mL of algal culture). Cells from strain 624 in their exponential growth phase were plated in 24- well plates at a final concentration of 10^5^ cell mL^-1^, and exposed to either live *P. piscicida* cells or filtrate. These experiments were conducted with both xenic and axenic cultures of *E. huxleyi*. Each treatment was replicated in triplicate. Prior to inoculation into 24-well plates, the bacteria were enumerated on a flow cytometer and diluted appropriately. Filtrate from the *P. piscicida* culture was obtained by filtering 5 mL of live culture through a 0.2 μm sterile syringe tip filter. Prior to sampling for phytoplankton cell abundance, triplicate wells were gently mixed via pipet and 50–200 μL aliquots were taken from each replicate, pipetted into a 96-well plate, and run on the flow cytometer. Growth rate was calculated by using the exponential growth equation,

$$\mathrm{Growth}\;\mathrm{rate}\;=\;\ln(A_{f}/A_{i})/T_{f}-T_{i}$$

where A is the abundance and T is the time, over the first 72 h of the experiment. Significant differences in growth rates of *E. huxleyi* in response to chemical additions were determined by using a one-way analysis of variance (ANOVA) and Tukey’s HSD *post hoc* analysis. All statistical analyses were performed using MatLAB (v. 8.3). Those treatments where the growth rate was significantly different from the algae only control (*p* < 0.05) were considered to have activity.

### Bioassay-Guided Fractionation of Exudates from *P. piscicida*

Assessment of semi-purified fractions via the growth inhibition assay was carried out using axenic *E. huxleyi* strain 624 to determine those algicidal compounds exuded by *P. piscicida* (**Supplementary Figure S1**). *E. huxleyi* (624) cells in exponential growth phase were plated at 10^5^ cell mL^-1^ in to 24-well plates in triplicate and exposed to crude and semi-purified fractions dissolved in DMSO, with DMSO concentrations not exceeding 0.2% v/v. Appropriate controls were run with each growth inhibition assay, i.e., f/2 media only, DMSO without compounds, were performed in triplicate. Well plates were incubated under general culturing conditions, and enumerated on the flow cytometer, as described above. At each step of the chemical fractionation process described below, semi-purified fractions were tested in the growth inhibition assay using axenic *E. huxleyi* strain 624 and included the addition of paired controls (TSY media only, f/2, and DMSO). Statistical comparison of growth rates resulting from semi-purified fractions were compared to controls as described in Section “Phytoplankton Growth Inhibition Assay.”

The entire crude extract (1260 mg; described above) was applied to a silica gel column and eluted with a step-gradient of isooctane, (4:1) isooctane/ethylacetate (EtOAc), (3:2) isooctane/EtOAc, (2:3) isooctane/EtOAc, (1:4) isooctane/EtOAc, EtOAc, (1:1) EtOAc/MeOH, and 100% MeOH, yielding eight fractions. A total of 458 mg of material eluting with (1:1) EtOAc/MeOH from the silica column was separated further by solid-phase extraction (Supelclean ENVI-18) eluting with a step-gradient of (20:1) water/acetonitrile, (4:1) water/acetonitrile, (3:2) water/acetonitrile, (2:3) water/acetonitrile, (1:20) water/acetonitrile, and acetone. All solvents, except acetone, were acidified with 0.1% formic acid. The active fraction, totaling 9.73 mg, eluted with (2:3) water/acetonitrile, and was separated further by semipreparative HPLC using an Agilent 1200 series HPLC and a Phenomenex Kinetex 5 μm C_18_ 100 Å (150 mm × 10 mm) column, heated to 30°C, and a gradient of 42–95% acetonitrile in water with a flow rate of 4 mL min^-1^. All solvents were acidified with 0.1% formic acid. Fractions were pooled according to UV adsorption characteristics at 276 nm. A single active fraction eluted in the 58% water/42% acetonitrile fraction. A total of 1 mg of the active compound, determined to be 2-heptyl-4-quinolone, was recovered from 10.5 L of culture after 8 days of growth, yielding a final concentration of 0.39 μM.

### Structure Determination of Bacterial Compounds

NMR experiments were conducted using an Agilent NMR 500 MHz (Agilent Technologies, Santa Clara, CA, USA) with DMSO-*d*_6_ as the solvent (referenced to residual DMSO at δ_H_ 2.50 and δ_C_ 39.5) at 25°C. HHQ standard was acquired from Sigma Aldrich (St. Louis, MO, USA).

Accurate mass spectra for 2-heptyl-4-quinolone was acquired on an Agilent Technologies 6230 TOF with a Dual Agilent Jet Stream Electrospray Ionization source, equipped with an Agilent 1260 Infinity series HPLC with a Phenomenex Kinetex 2.6 μm, C_18_, 100 Å, LC column (150 mm × 2.1 mm) as the stationary phase with a flow rate of 0.2 mL min^-1^. The ion source was operated at 350°C and 3500 V with a nitrogen gas flow of 8 L min^-1^, nebulizer pressure of 40 psi and fragmentor voltage of 135 V. The chromatrography method was as follows: 0–10 min at (1:1) water/acetonitrile; then ramped to (1:20) water/acetonitrile over the next 7 min; held at (1:20) water/acetonitrile for 3 min; then returned to (1:1) water/acetonitrile and held for 3 min. All solvents were acidified with 0.1% formic acid. The instrument was equipped with an Agilent Mass Hunter Workstation version B0.4.00 software.

### Bioinformatic Analysis of *P. piscicida* Genome

High purity *P. piscicida* genomic DNA was extracted, purified, and sequenced using the Ion PGM^TM^ Hi-Q Sequencing Kit according to the methods of Agarwal et al. (2014). Genomic DNA sequences were assembled into contigs using the SPAdes Genome Assembler (v 3.6.0; Bankevich et al., 2012), and the draft genome was annotated using RAST (Aziz et al., 2008). Following RAST annotation, a homology search was conducted for *P. piscicida* homologs matching the *Pseudomonas aeruginosa pqsABCDE* operon, *pqsR*, and *pqsH* genes, previously determined to be involved in alkylquinoline synthesis (Dulcey et al., 2013; Drees and Fetzner, 2015) using SEED Viewer (Version 2.0; Overbeek et al., 2005).

### Dose-Response Experiments

The dose-response relationship of 2-heptyl-4-quinolone was measured in *E. huxleyi* (strains 624, 374, 379), *D. tertiolecta*, and *P. tricornutum* in growth inhibition assays with pure compounds tested at various concentrations in triplicate. All the *E. huxleyi* strains were axenic, where as the strains of *D. tertiolecta* and *P. tricornutum* were xenic. Dose-response experiments were conducted similarly to the growth inhibition assays. Cells from exponentially growing cultures were grown in triplicate 24-well plates. Initial cell abundance was determined by carbon concentration (Menden-Deuer and Lessard, 2000), where each well had an initial carbon concentration of 1.2 × 10^6^ pg C mL^-1^. Well plates were kept under general culturing conditions, and cell abundance was monitored daily using a flow cytometer (Guava, Millipore) as described above.

Phytoplankton growth rate (μ d^-1^) was plotted against the concentration of each pure compound in order to determine the concentration of compound resulting in 50% growth inhibition (IC_50_). Pure compound IC_50_ values were calculated and 95% confidence intervals were estimated using Prism 6.0 software (GraphPad) by fitting the log transformation of the response variable (I; inhibitor concentration) by non-linear regression to the equation (1); where the slope factor (Hill Slope) is equal to -1.0 and the “Top” and “Bottom” numerals are equal the plateaus of curve in units of growth rate.

$$\left(1)\;Y\;=\;\mathrm{Bottom}\;+\;(Top-Bottom)/(1+10^{\left(({\mathrm{LogIC}}_{50}-I)*\mathrm{Hill}\;\mathrm{Slope}\right)}\right)$$

### Chemical Profiling and Phylogenetic Analysis of *Pseudoalteromonas* sp.

DNA sequencing and phylogenetic analysis of marine isolate B030a (GenBank No. KT804650) was performed as described in Whalen et al. (2015). Evolutionary history of *Pseudoalteromonas* isolates were inferred using the neighbor-joining methods and the optimal tree is shown for topology. Exuded metabolites from 37 *Pseudoalteromonas* isolates in our chemical library underwent untargeted metabolomic fingerprint analysis as described in Whalen et al. (2015). A list of chemical features [*m/z* – retention time (RT) pairs] was filtered to include only those features that had concentrations that were at least one order of magnitude higher in the *Pseudoalteromonas* sample than in the media blank. This filtered list of features was then searched for the corresponding ion and RT in (-)-HRESI mode matching the standard 2-heptyl-4-quinolone. Phylogenetic analysis was performed as described in Whalen et al. (2015) and a heat map was generated showing the relative abundances of 2-heptyl-4-quinolone in each *Pseudoalteromonas* isolate.

## Results

### Phytoplankton Response to Live Bacterial Cells and Crude Extract

**FIGURE 1:**
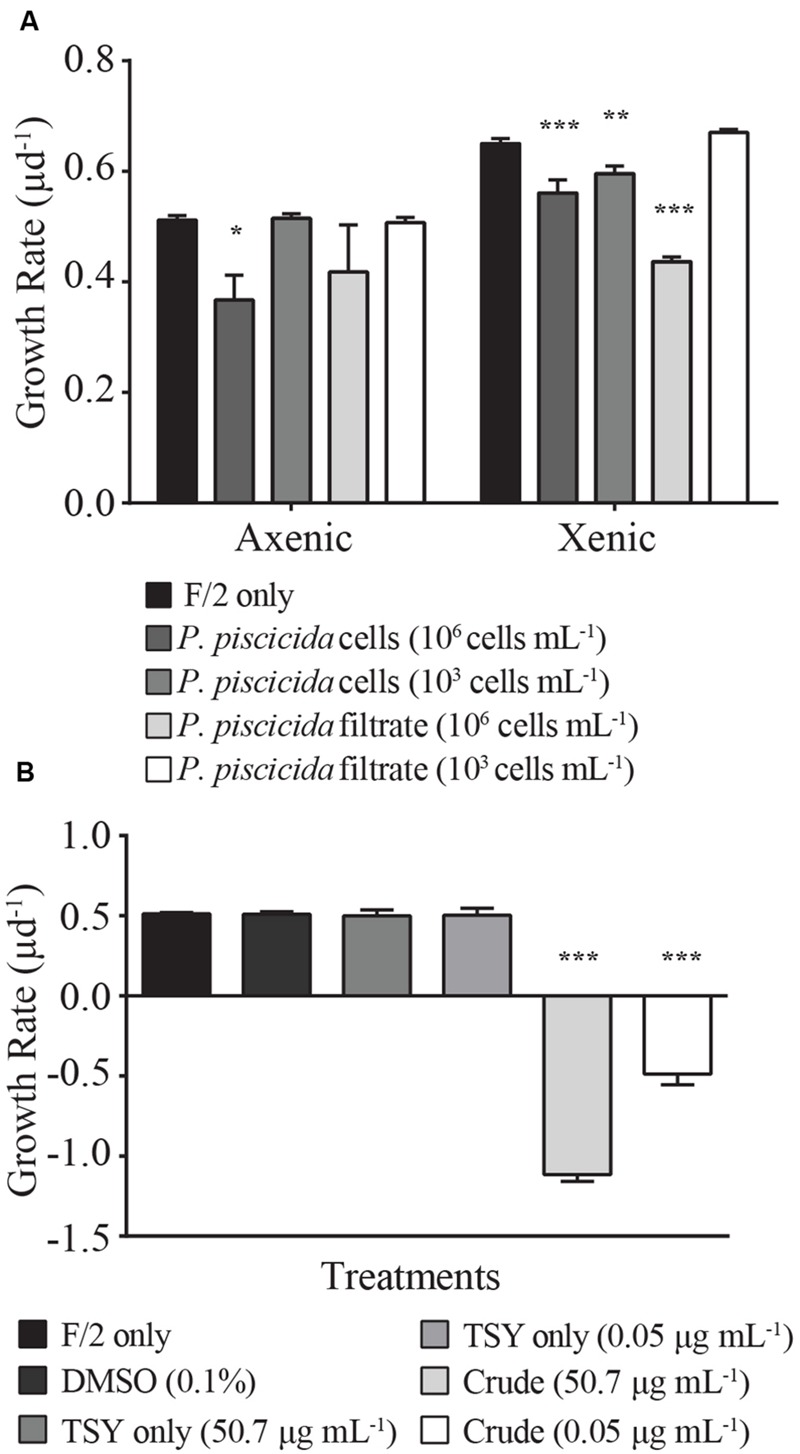
**Growth rates (μ d^-1^) of *Emiliania huxleyi* (strain 624, either xenic or axenic) were measured in response to **(A)** live *Pseudoalteromonas piscicida* cells and filtrate.** Growth rates (μ d^-1^) of axenic *E. huxleyi* (strain 624) were measured in response to **(B)** crude extracts of *P. piscicida* secreted metabolites and appropriate media (F/2 and TSY) and solvent (DMSO) controls. Compared to the control, the growth rate of *E. huxleyi* significantly decreased in response to high concentrations (10^6^ cells mL^-1^) of *P. piscicida* and the crude extracts. Additionally, high concentrations of culture filtrate and low concentrations (10^3^ cells mL^-1^) of *P. piscicida* resulted in a significant decrease in *E. huxleyi* growth but only in the xenic subculture. Significance is denoted as being less than 0.05 (^∗^), less than 0.01 (^∗∗^), or less than 0.001 (^∗∗∗^). For all treatments, *n* = 3. All error bars are one standard deviation of the mean.

The presence of live *P. piscicida* cells caused significant mortality in *E. huxleyi*. When cultures of *E. huxleyi* (strain 624) were exposed to “high” concentrations of *P. piscicida* cells (10^6^ cells mL^-1^), the growth rate of both axenic and xenic *E. huxleyi* cultures decreased 14 ± 5 and 28 ± 9%, respectively (**Figure 1A**; axenic, *p* = 0.015; xenic, *p* = 0.001). When exposed to “low” concentrations of *P. piscicida* cells at 10^3^ cells mL^-1^, only xenic *E. huxleyi* had a significantly depressed (6 ± 1%) growth rate (*p* = 0.0048); whereas, axenic *E. huxleyi* were unaffected compared to f/2 controls. Additionally, filtrate from *P. piscicida* cells at 10^6^ cells mL^-1^ significantly reduced the growth of xenic *E. huxleyi* by 32 ± 6% (*p* < 0.001).

Axenic *E. huxleyi* was exposed to crude extracts of exuded compounds produced by *P. piscicida* at a final concentration of 50.7 and 0.05 μg mL^-1^, concentrations approximately equivalent to the material exuded by 10^6^ and 10^3^ cells mL^-1^, respectively. Exposure to crude extracts resulted in significant mortality for *E. huxleyi* (**Figure 1B**; *p* < 0.001), with negative growth rates for *E. huxleyi* when exposed to either concentration of crude extract. Conversely, when exposed to crude extracts of TSY media, no significant difference in growth rate was observed relative to the DMSO and media only controls, indicating the causative agent of mortality was the result of a compound excreted by *P. piscicida*.

### Compound Identification and Metabolomic Fingerprinting

**FIGURE 2:**
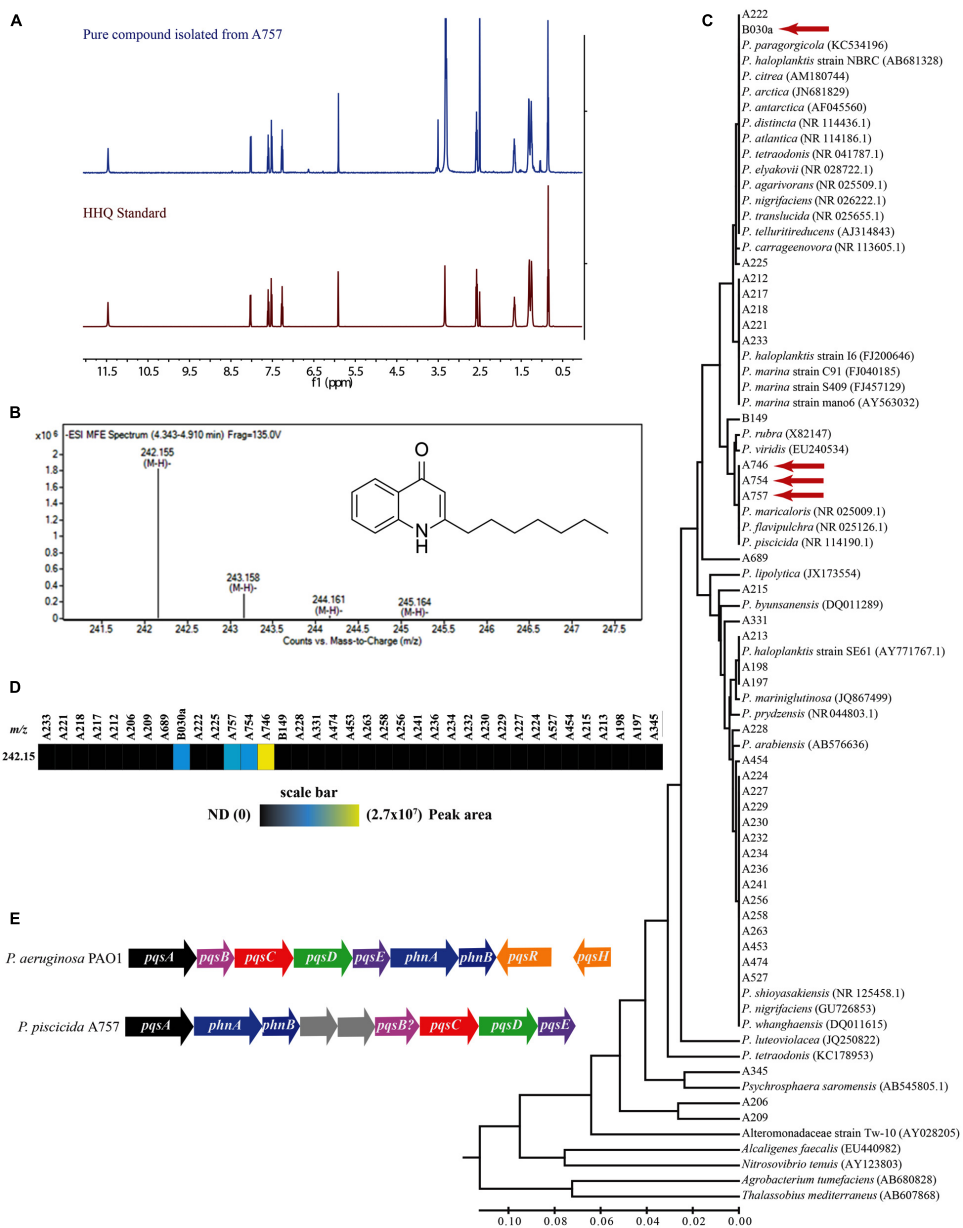
**Composite figure showing HHQ structural elucidation from isolate A757, chemophylogenetic analysis of HHQ producing *Pseudoalteromonas* sp., and HHQ biosynthetic machinery in *P. piscicida* (A757). (A)** Structural identification of 2-heptyl-4-quinolone (HHQ) from *P. piscicida.* ^1^H-NMR spectrum 500 MHz, DMSO-*d6* of the pure compound isolated from *P. piscicida* (blue) and the authentic standard HHQ (red). **(B)** Mass spectrum [(-)-HRESI] of the active constituent from *P. piscicida* (A757; structure of HHQ in inset). **(C)** Phylogenetic tree and comparison of HHQ production in 37 isolates of *Pseudoalteromonas* sp. The evolutionary history was inferred using the UPGMA method (Sneath and Sokal, 1973). The optimal tree with the sum of branch length = 0.83629248 is shown. The tree is drawn to scale, with branch lengths in the same units as those of the evolutionary distances used to infer the phylogenetic tree. The evolutionary distances were computed using the Maximum Composite Likelihood method (Tamura et al., 2004) and are in the units of the number of base substitutions per site. The analysis involved 77 nucleotide sequences. All positions containing gaps and missing data were eliminated. There were a total of 394 positions in the final dataset. Evolutionary analyses were conducted in MEGA6 (Tamura et al., 2013). Isolates identified to produce HHQ are indicated with a red arrow. **(D)** Heat map showing the relative abundance of HHQ produced by *Pseudoalteromonas* sp. profiled in Whalen et al. (2015). Colored bars indicate the peak area of HHQ for each marine isolate indicated by the column headings. **(E)** A comparison of the alkylquinoline biosynthetic pathway in *Pseudomonas aeruginosa* PAO1 and *Pseudoalteromonas piscicida* (A757; GenBank Accession numbers, KT879191–KT879199).

The secreted algicidal compound produced by *P. piscicida*, was isolated using bioassay-guided fractionation. The purified active fraction was investigated with 1D-NMR and ESIMS experiments. ^1^H and ^13^C NMR showed the presence of a seven carbon straight alkyl chain (δ_H_ 0.85, 1.26, 1.32, 1.67, and 2.58 and δ_C_ 13.9, 22.0, 28.3, 28.4, 28.5, 31.1, and 33.2) and a 1,2 substituted benzene ring [δ_H_ 7.26 (d), 7.52 (t), 7.60 (t), and 8.03 (d); **Figure 2A** and **Supplementary Figure S2**]. These data along with a robust negative ESIMS ion at 242.155 *m/z* (**Figure 2B**) strongly suggested the known bacterial metabolite 2-heptyl-4-quinolone (HHQ, C_16_H_21_NO; Bredenbruch et al., 2005). Comparison of ^1^H and ^13^C NMR spectra of the active compound to an HHQ standard confirmed that the chemical structures were identical. Untargeted metabolomic profiling of *Pseudoalteromonas* crude extracts as detailed in Whalen et al. (2015), indicated HHQ was present in isolates A757, A754, A746, and B030a (**Figures 2C,D**). The production of HHQ is the most pronounced in a clade containing A757, A754, and A746 representing a distinct chemotype characterized by halogenation of the exometabolome (Whalen et al., 2015). 16S rDNA sequencing indicates A757, A754, and A756 are all *P. piscicida* and were isolated from plastic debris in the North Atlantic (Whalen et al., 2015). The HHQ-producing marine isolate B030a was isolated from macroalgae along the coast of Woods Hole, MA, USA, but is not in the same clade as A757, A754, and A746, rather this B030a falls within a large clade represented by diverse group of *Pseudoalteromonas* sp. (**Figure 2C**).

### Identification of HHQ Biosynthetic Machinery in *P. piscicida*

The draft genome sequence of *P. piscicida* was determined to be 5.1 Mbp in size with an estimated 4543 coding sequences. The *P. piscicida* genome was mined using SEED Viewer for the identification of genes involved alkylquinoline synthesis (GenBank Accession numbers, KT879191–KT879199). In *P. aeruginosa*, quorum sensing is controlled by the transcriptional regulators LasR, RhIR, and MvfR (PqsR). The former two are controlled by homoserine lactones, while the signaling molecules that activate MvfR, a LysR-type transcriptional regulator, belong to a family of 4-hydroxy-2-alkylquinolines (HAQs), including 3,4-dihydroxy-2-heptylquinoline (PQS, 258.149 *m/z* [M-H]), its direct precursor 2-heptyl-4-quinolone (HHQ), and 4-hydroxy-2-heptylquinoline-*N*-oxide (HQNO; Dulcey et al., 2013). The presence of a single homolog to each of the *P. aeruginosa pqsA, pqsC, pqsD*, and *pqsE* genes were identified in the *P. piscicida* genome, with amino acid identities of 37.9, 28.5, 47.2, and 30.1%, respectively. However, *in silico* analysis indicated the operon arrangement was different to that of *P. aeruginosa* (**Figure 2E**). The coding sequence with the highest similarity to *P. aeruginosa pqsB* was positioned immediately upstream of the homolog *pqsC* in the *P. piscicida* genome with 20.3% amino acid identity. The position of this hypothetical protein within the operon containing other *pqsABCDE* homologs would suggest it is a possible homolog to *pqsB*. The genome of *P. aeruginosa* also encodes two anthranilate synthases, PhnA and PhnB, immediately downstream of the *pqsABCDE* operon, which supply anthranilic acid. The genome of *P. piscicida* contains two homologs of PhnA and PhnB (**Figure 2E**) with amino acid identities of 29.8 and 40.5%, respectively; however, their position is shifted in comparison to *P. aeruginosa* and downstream of the homolog to *pqsA*.

After finding the *pqsABCDE* homologs in our *P. piscicida* (A757) isolate, we were interested in determining if this operon exists in other *Pseudoalteromonas* sp. whose genome has been made publically available. Using the Department of Energy – Joint Genome Institute’s Integrated Microbial Genome (IMG) system, we searched 67 *Pseudoalteromonas* sp. available genomes that were categorized as finished, in permanent draft, or draft stage against two *P. aeruginosa* PAO1 genomes (637000218 and 2623620964) for *pqsABCDE* operon homologs. Using a minimum identity cutoff of 10% and e-value of 0.01, we were able to find representatives of many of the genes in the pqs operon (**Supplementary Figure S3A**). However, upon closer manual inspection of these genomes, only *Pseudoalteromonas citrea* NCIMB 1889 maintained the synteny of the pqs operon as seen in *P. piscicida* (A757; **Supplementary Figure S3B**).

### Dose-Response Experiments with Various Phytoplankton Species

**FIGURE 3:**
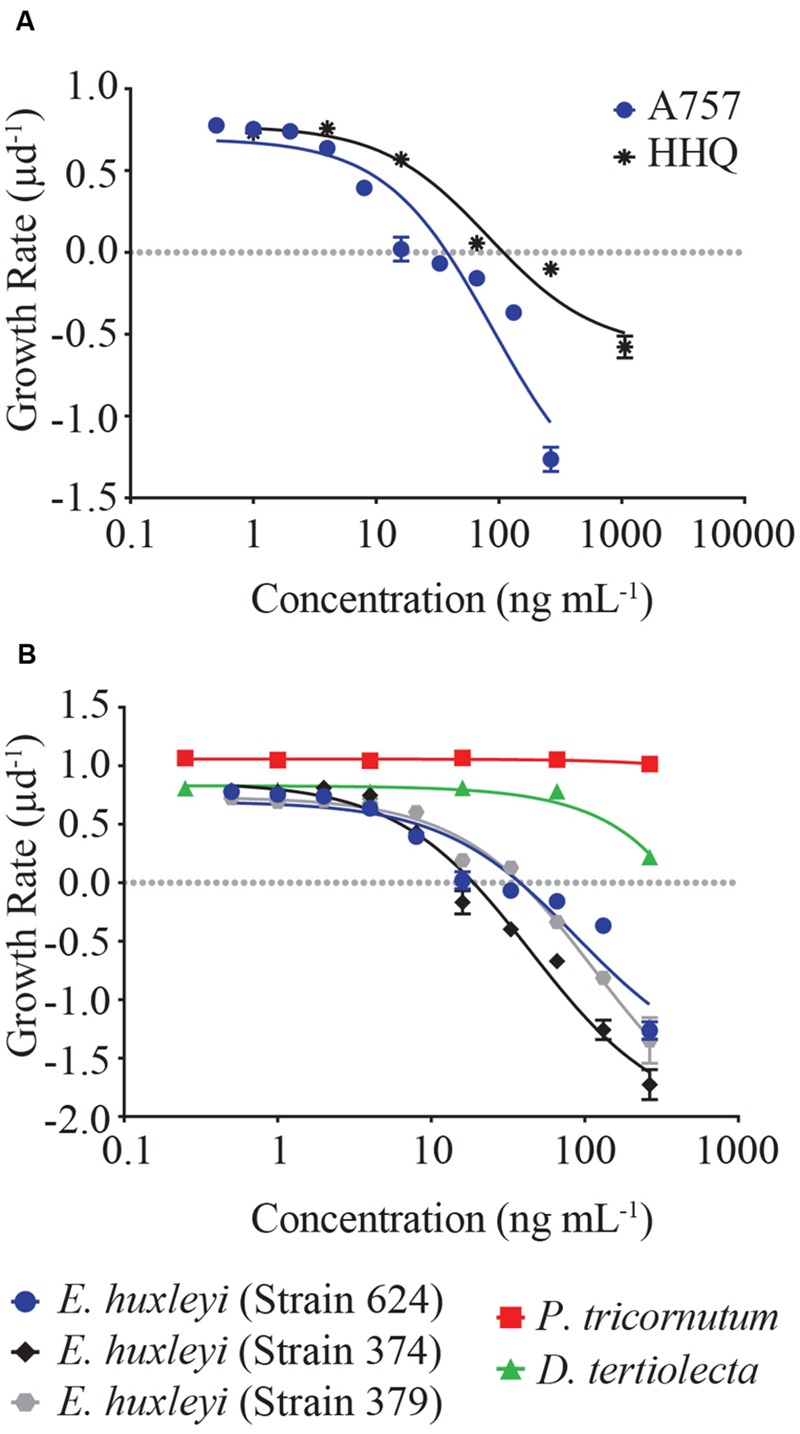
**(A)** Dose-response comparison of *E. huxleyi* (strain 624, axenic) growth rate in response to a gradient of extracted (labeled A757) and reference HHQ. **(B)** Dose-response curves of five different strains of phytoplankton in response to HHQ extracted from *P. piscicida*. Each symbol represents the mean of three independent replicates ± the standard deviation. IC50 values were calculated for each curve (see **Table 1**).

Dose-response experiments with axenic *E. huxleyi* (strain 624) determined the IC_50_ concentration for the isolated pure compound to be 88.3 ng mL^-1^, which was not significantly different (*p* = 0.92) from the IC_50_ obtained when exposed to the purchased HHQ standard (**Figure 3A**, **Table 1**; Sigma-Aldrich). For all three *E. huxleyi* strains, IC_50_ concentrations for the isolated pure compound ranged from 44.51 to 115.4 ng mL^-1^ (**Figure 3B**; **Table 2**). IC_50_ values were found to differ significantly (*p* = 0.003) when all three strains of *E. huxleyi* were compared. The naked strain, 374 was found to be the most sensitive to HHQ, having the lowest IC_50_ value, while the haploid strain, 379 was the least sensitive. A comparison of IC_50_ values obtained for all three strains of *E. huxleyi* with other phytoplankton species, *D. tertiolecta* and *P. tricornutum*, indicated *E. huxleyi* was significantly more susceptible to HHQ, despite both *D. tertiolecta* and *P. tricornutum* being vulnerable to the crude *P. piscicida* extract (**Supplementary Figure S4**).

**Table 1:** Statistical comparison of the dose-response curves and IC_50_ values generated for *Emiliania huxleyi* (Strain 624) exposed to pure compound from A757 and the HHQ standard.

Compound	IC_50_ (ng mL^-1^)	nM	*R*^2^	95% CI	*p*-value
A757	88.31	362	0.91	45.7–170.4	0.9218
HHQ standard	83.07	341	0.96	50.0–138.0	

**Table 2:** Inhibitory concentration (IC_50_) of HHQ isolated from *Pseudoalteromonas piscicida* in five different strains of phytoplankton.

Species	IC_50_ (ng mL^-1^)	nM	*R*^2^	95% CI
*Emiliania huxleyi* (624)	88.31	362	0.91	45.7–170.4
*E. huxleyi* (374)	44.51	182	0.98	33.3–59.4
*E. huxleyi* (379)	115.4	474	0.98	87.2–152.8
*Dunaliella tertiolecta*	>100000	–	–	–
*Phaeodactylum tricornutum*	>100000	–	0.93	–

**FIGURE 4:**
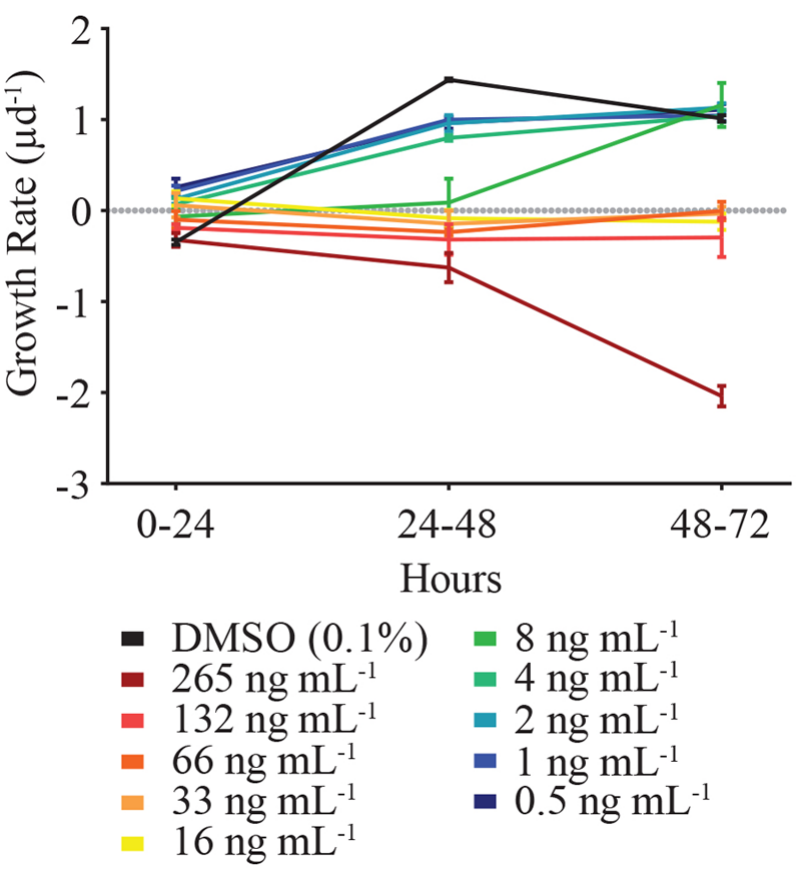
**The incremental growth rate (μ d^-1^) of *E. huxleyi* (strain 624) over the experimental time period.** At toxic concentrations (between 8 ng mL^-1^ and above), *E. huxleyi* growth rate remained relatively consistent among the time periods examined. Thus, the presence of HHQ results in static *E. huxleyi* growth rather than cell lysis. Error bars are one standard deviation of the mean.

In order to decipher when HHQ-induced mortality of *E. huxleyi* (strain 624) occurred, growth rates of the alga were examined across 24 h time increments (**Figure 4**). While the growth rate of *E. huxleyi* when exposed to HHQ concentrations was dose-dependent, daily growth rate in many of the concentrations remained relatively stable over time. For example, daily growth rate when exposed to 132 ng mL^-1^ of HHQ ranged from -0.19 to -0.32 d^-1^, over the experimental time period. This indicates that the total loss of *E. huxleyi* when exposed to high concentrations of HHQ occurs over time, rather than a singular loss event.

## Discussion

### Bacterially Mediated Mortality of Marine Phytoplankton

Quorum sensing molecules mediate bacterial cell–cell communication allowing cells to respond collectively to changes in their environment. This study is the first to identify a quorum sensing precursor molecule that induces mortality in marine phytoplankton. Initial culturing experiments with high *P. piscicida* cell densities (10^6^ cells mL^-1^) resulted in significant mortality in *E. huxleyi* cultures. This finding is concurrent with previous observations demonstrating high concentrations of *Pseudoalteromonas* sp. induced mortality in a wide range of phytoplankton species including raphidophytes, gymnodinoids, and diatoms (Lovejoy et al., 1998; Mitsutani et al., 2001). While the concentration of bacteria used in these experiments is high compared to natural abundances, bacterial behavior (e.g., swarming, biofilm formation) can create localized concentrations of bacteria on or around phytoplankton cells. Furthermore, in some instances, it is only when bacteria are attached to surfaces that they produce active metabolites, thereby generating microenvironments of high metabolite concentrations (Long et al., 2003).

Algicidal activity can be mediated either directly (bacterial cell – phytoplankton contact) or indirectly via chemical interactions (**Figure 1**). Filtrate from *P. piscicida* cells resulted in *E. huxleyi* mortality, indicating a possible chemical basis for the algicidal activity observed. Bio-assay guided fractionation revealed that HHQ, a compound released by *P. piscicida*, caused inhibition of *E. huxleyi* growth. This study is the first observation of HHQ production by a marine *Pseudoalteromonas* species. Previous work describes PQS and HHQ production as occurring during the stationary phase of growth, and both can function as antibiotics and signaling molecules in cell–cell communication, with roles in virulence, apoptosis, and viability of fungal and mammalian cells (Bredenbruch et al., 2005; Calfee et al., 2005; Reen et al., 2011). Unlike PQS, which has poor solubility in water; HHQ can passively diffuse into the external aqueous environment (Deziel et al., 2004). These diffusible signaling molecules, termed autoinducers, are a form of intercellular communication released by bacteria in a cell density-dependent manner, designed to engage cells in cooperative and coordinated behavior beneficial to the entire bacterial population. Once these autoinducers reach critical threshold, they cause the transcriptional activation of quorum-sensing-controlled genes that are key in biofilm formation, virulence factor production (Bredenbruch et al., 2005), secondary metabolite production, motility (Reen et al., 2011), and pathogenesis (Zaborin et al., 2009).

Exposure to HHQ resulted in a significant decrease in growth rate and eventual mortality of *E. huxleyi* (**Figures 3** and **4**). While this is the first observation of HHQ-induced mortality in a phytoplankton species, responses to other alkyl-quinolones have been documented in phytoplankton. For example, Long et al. (2003) found that exposure to 2-*n*-pentyl-4-quinolinol (PQ), also produced in the PQS pathway, resulted in mortality of several phytoplankton species. These results imply that HHQ and other compounds produced in the PQS pathway may have functions beyond quorum sensing and anti-microbial activity, and function as inter-domain cues that initiate eukaryotic cell death. While the concentration of HHQ in the marine environment has not been measured, acyl-homoserine lactones (AHLs), another class of autoinducers, have been measured at nanomolar concentrations in the aqueous phase or higher in biofilms (Wheeler et al., 2006) and have been implicated in the control of degradation of sinking detritus (Hmelo et al., 2011) and involved in phosphorus acquisition by epibonts of *Trichodesmium* sp. (Van Mooy et al., 2012). The IC_50_ observed for *E. huxleyi* in response to HHQ is within the nanomolar range (**Tables 1** and **2**), implying that if the concentration of HHQ parallels other quorum sensing molecules in the marine environment, especially within the context of a biofilm, *E. huxleyi* would be highly susceptible to this autoinducer.

It is worth noting that HHQ was not toxic to all phytoplankton. When *D. tertiolecta* and *P. tricornutum* were exposed to HHQ, no significant change in growth rate was observed, even at high concentrations (**Figure 3**). This specificity in phytoplankton susceptibility to bacterial cells has been observed previously in response to the presence of live *Pseudoalteromonas* sp. cells (Lovejoy et al., 1998), indicating differential species sensitivity resulting from variability in chemical resistance mechanisms or target-site insensitivity. Interestingly, when *D. tertiolecta* and *P. tricornutum* were exposed to extracts of *P. piscicida* exudate, both phytoplankton species exhibited significant mortality in comparison to controls, suggesting this bacterium may produce a cocktail of algicidal compounds specific to different phytoplankton species.

### Phylogenetic Evaluation of HHQ Biosynthetic Pathways

Our survey of HHQ production in the *Pseudoalteromonas* sp. within our chemical library indicated that two clades contained representatives capable of producing HHQ at measureable levels in culture. Isolates A757, A754, A746, and B030a fall out by 16S rDNA into two clades that contain 19 different *Pseudoalteromonas* sp. HHQ-producing clades represent both open ocean and coastal species isolated from both abiotic and biotic surfaces, indicating HHQ-producing species can inhabit diverse marine environments. With the recent re-evaluation of the alkylquinoline biosynthetic pathway described from the pathogen *P. aeruginosa* in Dulcey et al. (2013) and Drees and Fetzner (2015), we identified homologs of the *pqsABCDE* operon responsible for HHQ synthesis in *P. piscicida* A757. PQS and HHQ are able to bind MvfR (PqsR) and induce the expression of the *pqsABCDE* operon, which controls biosynthesis of HAQs (Deziel et al., 2004; Dubern and Diggle, 2008). The presence of PqsA, anthranilate-CoA ligase, activates anthranilic acid into anthraniloyl-CoA which reacts with PqsD, a 3-oxoacyl synthase, to produce the intermediate, anthraniloyl-PqsD that is further modified by the synthases, PqsB, PqsC (Dulcey et al., 2013). The product of the gene *pqsE* is thought to be a thioesterase, hydrolyzing the biosynthetic intermediate 2-aminobenzoylacetyl-coenzyme A to form 2-aminobenzoylacetate, the precursor of HHQ (Drees and Fetzner, 2015).

We did not find PQS in our metabolomic analysis of *Pseudoalteromonas* sp. excretome, which is supported by the lack of a conclusive *P. piscicida* homolog to *pqsH*, the monooxygenase responsible for converting HHQ to PQS. The loss of the *pqsH* has been observed in two *Burkholderia* sp., which lacked PQS production, but produced HHQ (Diggle et al., 2006). The lack of an ion corresponding to PQS in *P. piscicida* A757 was confirmed by our untargeted metabolomic fingerprint analysis (Whalen et al., 2015). In *P. aeruginosa*, the LysR transcriptional regulator MvfR (PqsR) binds to the promoter of the *pqsABCDE* and is induced by HAQs. *P. piscicida* does contain a putative *pqsR* homolog identified as a LysR family transcriptional regulator with 23.2% similarity and located 2.1 Kbp away from *psqE*.

An analysis of the presence of this pathway in the publically available genomes of marine Pseudoalteromonads indicated that other species in this genus, including *P. citrea*, would have the biosynthetic capacity to produce HHQ. Interestingly, the mining of two additional *P. piscicida* publically available genomes (JGI: 2519899641 and 2541047159) indicated the lack of the pqs operon, suggesting the loss or reshuﬄing of these genes in subspecies. A recent study also indicated that the lack of PqsE homologs might not prohibit the synthesis of HHQ, rather the thioesterase function could be taken over to an extent by broad-specificity thioesterases, like TesB (Drees and Fetzner, 2015). This finding is significant to our study, as we generally lacked identifying homologs to *pqsE* in our genomic analysis.

In addition, other marine members of the gamma-proteobacteria have also been described to produce HHQ (Wratten et al., 1977; Debitus et al., 1998), including *P. aeruginosa*. Indeed, we confirmed the production of HHQ by comparison to the authentic standard via LC–MS from a marine isolate of *P. aeruginosa* (B323) in the Mincer Culture Collection isolated from water samples 430 km north of the British Virgin Islands (data not shown). These data indicate a more phylogenetically diverse grouping of marine members of the gamma-proteobacteria have the ability to produce algicidal compounds, including HHQ, consistent with earlier predictions of algicidal clades (Mayali and Azam, 2004); however, molecules responsible for the bioactivity have yet to be described. Additionally, Machan et al. (1992) reported HHQ inhibited the growth of *Staphylococcus aureus* and other gram-positive bacteria. While this finding was reported in clinical isolates of *P. aeruginosa*, an additional role of HHQ other than mediating cell–cell communication and phytoplankton abundance could include mediating Gram-positive bacterial populations, possibly associated with phytoplankton surfaces.

### Mode of Mortality and Biogeochemical Implications

Exposure to HHQ resulted in static growth of *E. huxleyi*, which eventually led to mortality of the alga, rather than an immediate cell lysis event upon exposure (**Figure 4**). Static growth upon exposure to HHQ has been observed in bacteria, with growth inhibition observed in several *Vibrio* species and a marine sponge bacterial isolate (Reen et al., 2011). In contrast, other studies have observed phytoplankton that were exposed to *Pseudoalteromonas* sp. and lysed within several hours of exposure (Lovejoy et al., 1998), indicating multiple compounds are acting through different mechanisms of action. We did identify a second compound from A757 exudate whose activity results in immediate cell lysis; however, attempts to identify this compound are still ongoing. Cell lysis verses static growth is an important distinction, as it ultimately impacts the fate of algal cells and carbon cycling. With static growth, *E. huxleyi* cells would remain in the water column, which could increase aggregation and export. Conversely, cell lysis is a final fate for the alga, fueling the microbial loop and decreasing carbon flow to higher trophic levels.

These results suggest that pelagic bacteria are not passive receivers of dissolved organic material. For example, it has been observed that when co-cultured under low iron conditions, *P. aeruginosa* cells lysed *Staphylococcus aureus* for its useable iron. Similar relationships have been proposed for bacteria–phytoplankton interactions, with bacteria having the potential to control algal blooms (Doucette et al., 1998). When the concentration of bacteria is high enough to induce quorum sensing (and the production of HHQ) the bacterial population could use the nutrients regenerated from static and dead phytoplankton cells to maintain growth. Algal substrates can provide ecological niches for species-specific bacterial populations to thrive (Teeling et al., 2012). These surface-associated bacteria may be relatively impervious to changes in seawater chemistry (*p*CO_2_); however, their population dynamics are tightly coupled to phytoplankton bloom development (Allgaier et al., 2008). Even a small increase in the exponential growth rate of a bacterial population can have large consequences for overall population abundance. Our results, while preliminary, provide novel hypotheses for further inquiry into the role of antagonistic phytoplankton-bacterial interactions. Furthermore, identification of the chemical compounds responsible for phytoplankton mortality will provide an enhanced ability to project shifts in population dynamics of both organisms.

The ability of HHQ to modulate interspecies interactions and cross-kingdom behavior (i.e., fungi and animals) as seen here and other studies (Reen et al., 2011), implicates alkylquinolone-signaling molecules as having important ecological roles in regulating primary production and affecting phytoplankton successions. HHQ could potentially alter both bacterial and phytoplankton composition, and physiologic parameters. Elucidating the impacts of this pathway will enable us to better parameterize phytoplankton population dynamics, and ultimately, provide better understanding of planktonic food web structure and biogeochemical cycling.

## Author Contributions

EH and KW designed experiments; KW performed the chemical isolation; EH performed phytoplankton growth assays; RD and DR solved the chemical structure; AE, MS, BM performed genome sequencing and assembly; EH and KW analyzed data; all authors participated in writing and editing manuscript.

## Conflict of Interest Statement

The authors declare that the research was conducted in the absence of any commercial or financial relationships that could be construed as a potential conflict of interest.

## Supplementary Material

FIGURE S1 **Flow chart detailing the bioassay guided fractionation process that was used to eventually identify HHQ as a causative compound influencing growth of *E. huxleyi***.

FIGURE S2 **Structural identification of 2-heptyl-4-quinolone (HHQ) from *P. piscicida*. ^13^C-NMR spectrum (75 MHz, DMSO-*d_6_*) of the pure compound isolated from *P. piscicida* (blue) and the authentic standard HHQ (red)**.

FIGURE S3 **Genome mining of pqs operon in *Pseudoalteromonas* sp. **(A)** The *pqs* operon from two species of *Pseudomonas aeruginosa* PAO1 were used to query 67 publically available *Pseudoalteromonas* sp. genomes.** Joint Genome Institute (JGI) genome accession numbers are listed for each isolate in parentheses. Red boxes indicate the presence of a homolog and black boxes indicate no homolog identified with BLAST cutoffs set at ≥10% identity and a maximum e-value of 0.01. White asterisks indicate two homologs (*pqsB* and *pqsE*) from *P. citrea* that were initially identified as absent in the genome using the Integrated Microbial Genome (IMG) gene-genome comparison analysis, but upon manual inspection, were found to be present. **(B)** A comparison of the alkylquinoline biosynthetic pathway in *Pseudoalteromonas piscicida* (A757; GenBank Accession no., KT879191–KT879199) and *P. citrea* (NCIMB 1889). Shading of homologs in *P. citrea* indicates percent amino acid identity to *P. piscicida* (A757) genes.

FIGURE S4 **Growth rate (μ d^-1^) for three species of phytoplankton exposed to the crude extract of the secreted metabolites of *P. piscicida*.** Despite HHQ having little effect on *D. tertiolecta* and *P. tricornutum*, toxicity to the crude extract of *P. piscicida* is observed in all species, indicating that *P. piscicida* likely produces additional compounds that result in algal mortality. Error bars are one standard deviation from the mean.
