# Supplementary figures and images for: A Bacterial Quorum-Sensing Precursor Induces Mortality in the Marine Coccolithophore, Emiliania huxleyi
Source: Front Microbiol. 2016 Feb 3;7:59. doi: 10.3389/fmicb.2016.00059 (PMC4737879; doi:10.3389/fmicb.2016.00059)

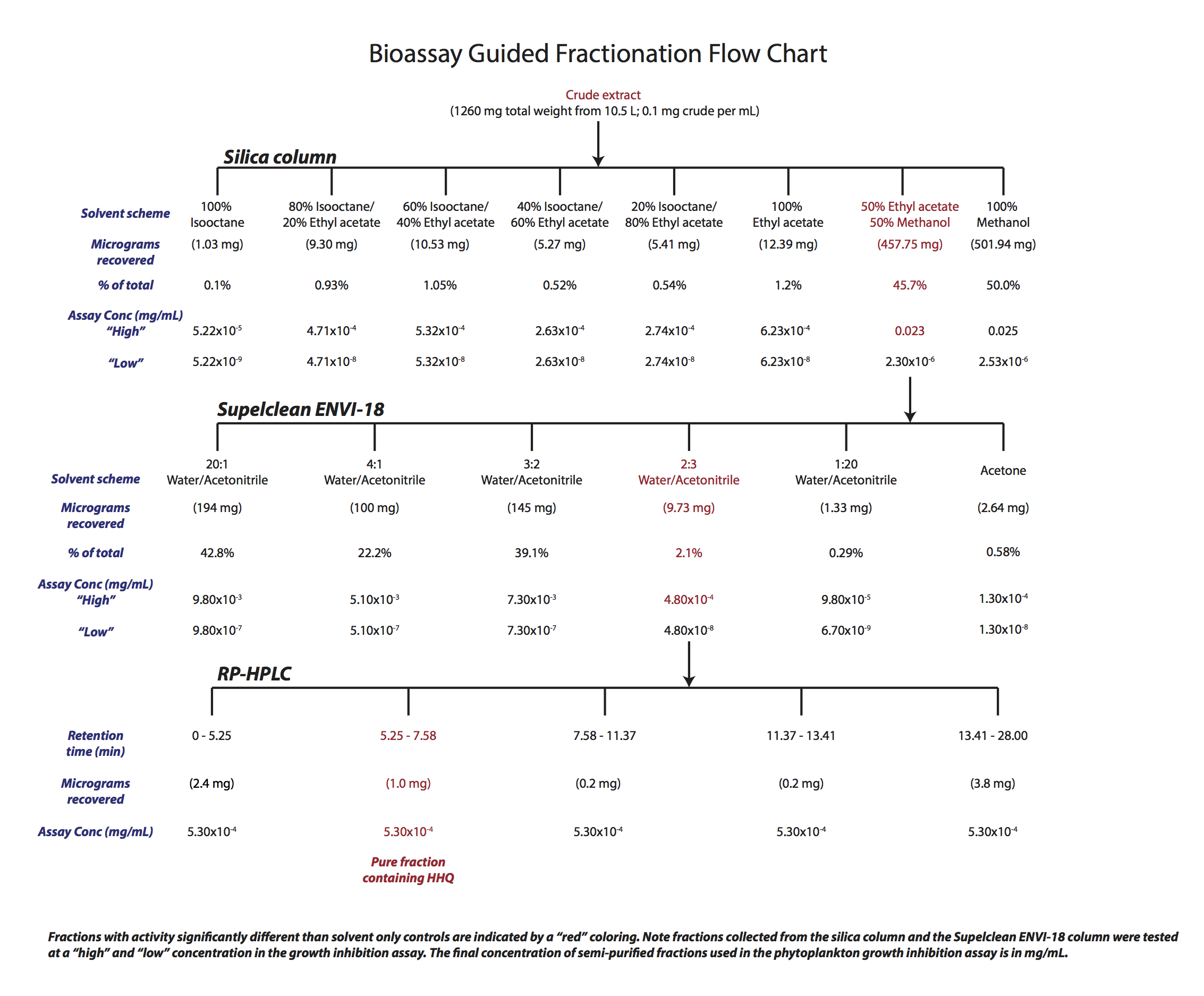

Supplement: FIGURE S1 — Flow chart detailing the bioassay guided fractionation process that was used to eventually identify HHQ as a causative compound influencing growth of E. huxleyi. [file Image_1.JPEG]

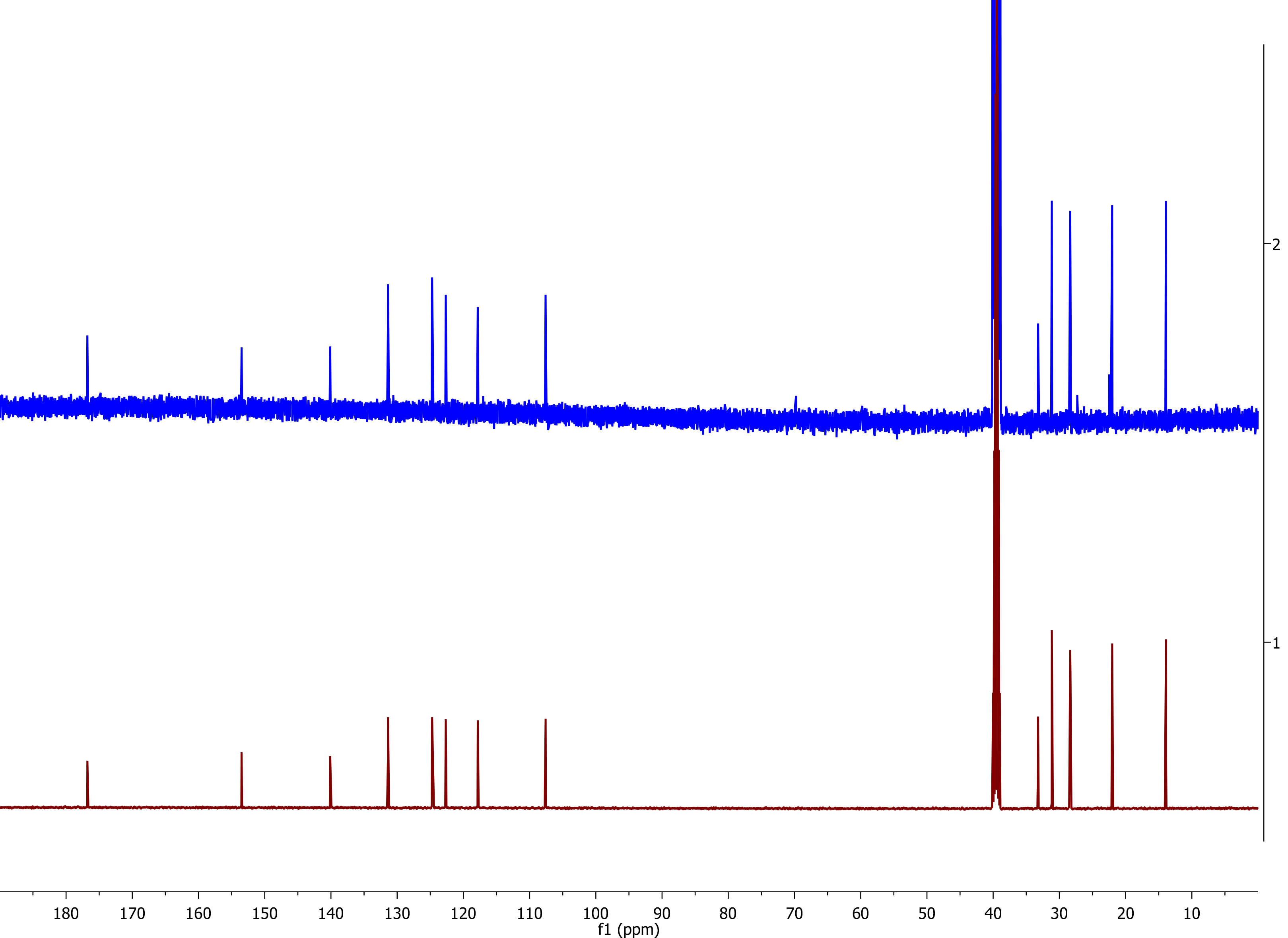

Supplement: FIGURE S2 — Structural identification of 2-heptyl-4-quinolone (HHQ) from P. piscicida. 13C-NMR spectrum (75 MHz, DMSO-d6) of the pure compound isolated from P. piscicida (blue) and the authentic standard HHQ (red). [file Image_2.JPEG]

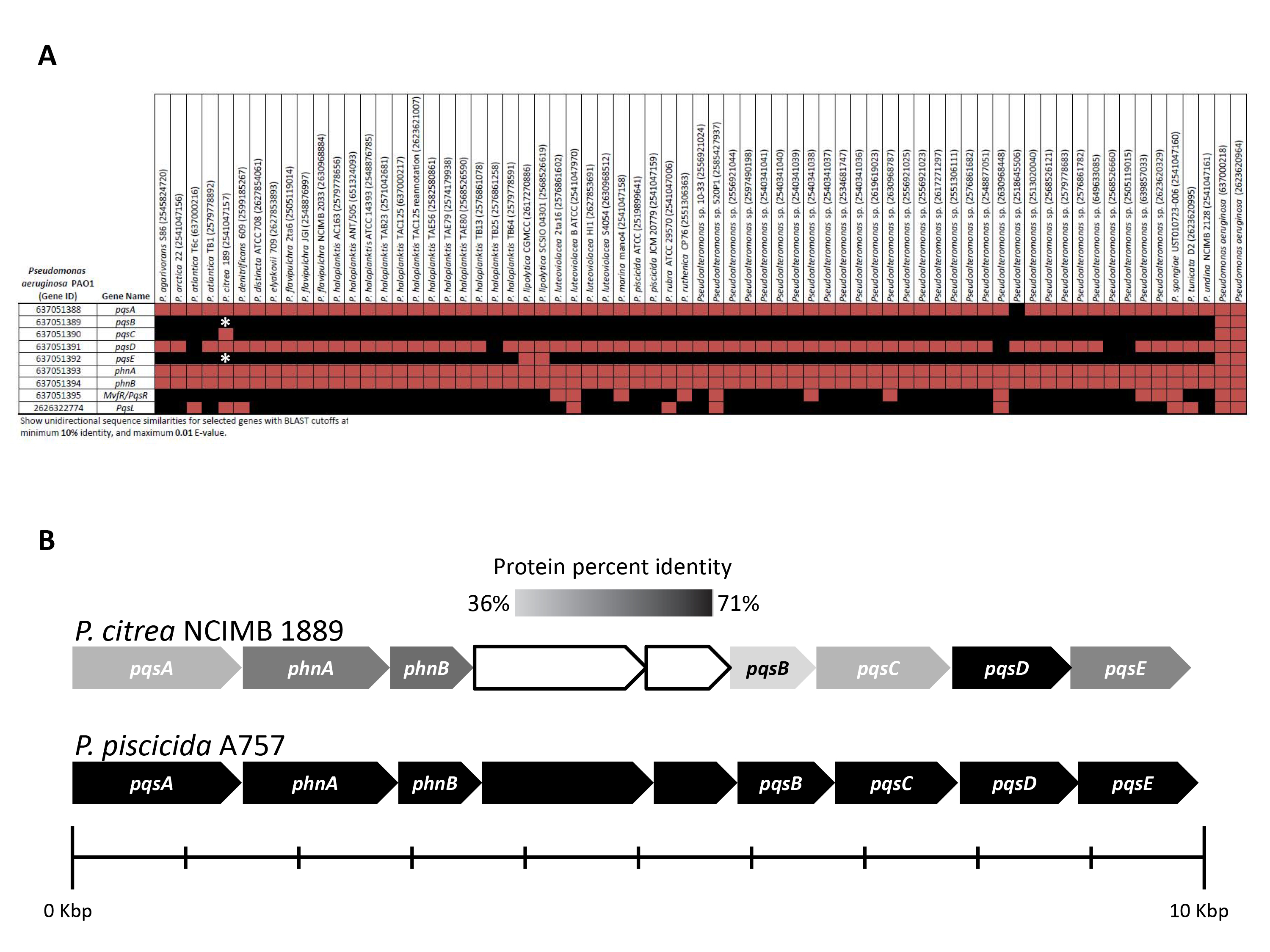

Supplement: FIGURE S3 — Genome mining of pqs operon in Pseudoalteromonas sp. (A) The pqs operon from two species of Pseudomonas aeruginosa PAO1 were used to query 67 publically available Pseudoalteromonas sp. genomes. Joint Genome Institute (JGI) genome accession numbers are listed for each isolate in parentheses. Red boxes indicate the presence of a homolog and black boxes indicate no homolog identified with BLAST cutoffs set at ≥10% identity and a maximum e-value of 0.01. White asterisks indicate two homologs (pqsB and pqsE) from P. citrea that were initially identified as absent in the genome using the Integrated Microbial Genome (IMG) gene-genome comparison analysis, but upon manual inspection, were found to be present. (B) A comparison of the alkylquinoline biosynthetic pathway in Pseudoalteromonas piscicida (A757; GenBank Accession no., KT879191–KT879199) and P. citrea (NCIMB 1889). Shading of homologs in P. citrea indicates percent amino acid identity to P. piscicida (A757) genes. [file Image_3.JPEG]

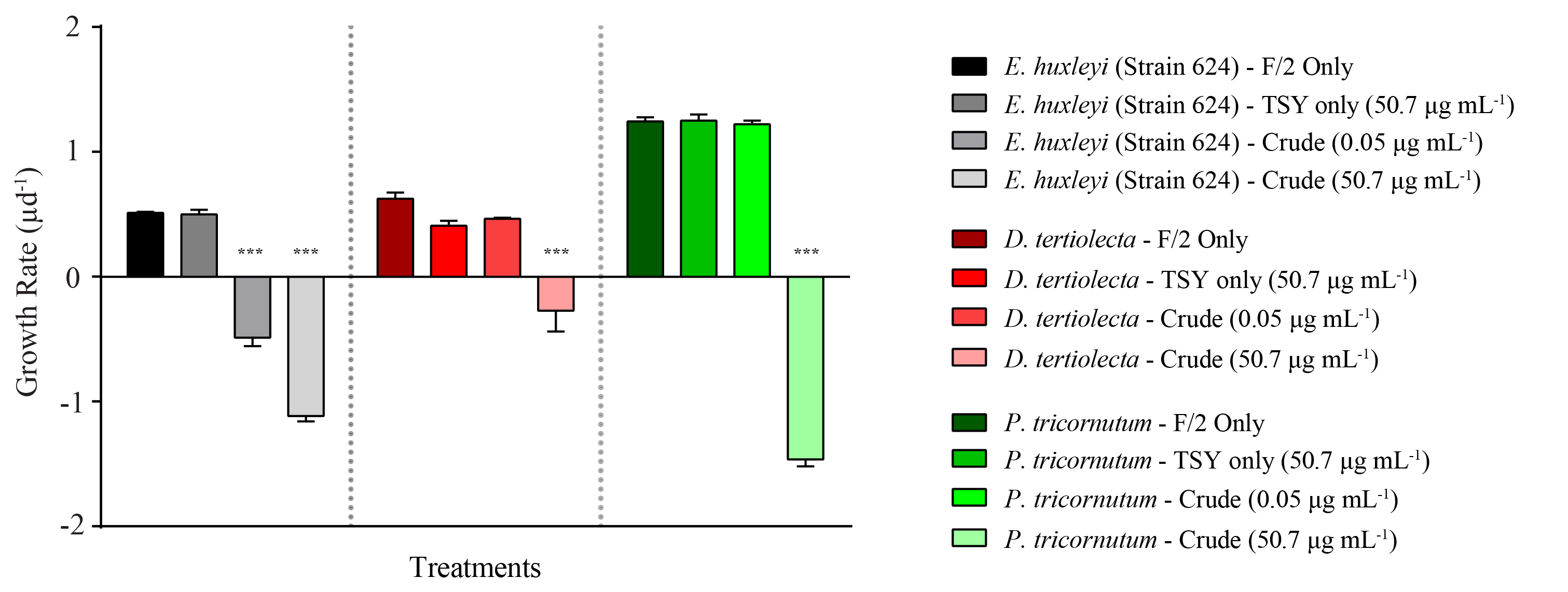

Supplement: FIGURE S4 — Growth rate (μ d-1) for three species of phytoplankton exposed to the crude extract of the secreted metabolites of P. piscicida. Despite HHQ having little effect on D. tertiolecta and P. tricornutum, toxicity to the crude extract of P. piscicida is observed in all species, indicating that P. piscicida likely produces additional compounds that result in algal mortality. Error bars are one standard deviation from the mean. [file Image_4.JPEG]
